# Editorial: The role of nutrition in healthy aging

**DOI:** 10.3389/fmed.2023.1335119

**Published:** 2023-12-04

**Authors:** Roberta Zupo, Fabio Castellana, Hélio José Coelho Júnior, Giovanni De Pergola, Maria Lisa Clodoveo, Rodolfo Sardone

**Affiliations:** ^1^Department of Interdisciplinary Medicine (DIM), University of Bari Aldo Moro, Bari, Italy; ^2^Department of Experimental Medicine, Section of Medical Pathophysiology, Food Science and Endocrinology, Sapienza University of Rome, Rome, Italy; ^3^Department of Biomedical Science and Human Oncology, School of Medicine, University of Bari Aldo Moro, Bari, Italy; ^4^Unit of Statistics and Epidemiology, Local Health Authority of Taranto, Taranto, Italy

**Keywords:** aging, nutrition, public health, epidemiology, diet

All over the world, people are living longer. According to the World Health Organization (WHO), by 2050 people aged 65 and older will account for nearly 17 percent of the population (1). While health research in older people generally focuses on the occurrence of multiple chronic diseases, significant health conditions and geriatric syndromes are prevalent and have their own multifactorial biological phenotypes that do not necessarily conform to discrete disease categories. In fact, aging is usually associated with a series of physiological changes covering a wide range of functional domains, manifested in outcomes of sarcopenia (2), frailty (3), malnutrition by defect or excess, as well as reduced quality of life, and many other declines found in elders. Diet, energy balance, nutrient intake, and lifestyle (4, 5) are modifiable factors (6) for which research efforts have yet to shed light in order to promote healthy aging, improve the quality of life in old age, and reduce the health burden of the elderly population. This Research Topic aimed to address this current issue in order to provide additional evidence able to fill the still-existing research gap on the biological mechanisms underlying the link between nutrition and healthy aging.

The collection included nine articles, mostly original, with a minority of two systematic review reports. The original research focused on the relationship between nutritional status and multiple health outcomes, such as survival, quality of life, heart failure, kidney illness, and cognitive impairment. Here, Franz et al. examined prospective data from the multicenter EMAAge cohort to assess the nutritional status of elderly patients with hip fractures, factors associated with malnutrition risk, and the association between malnutrition and mortality at 6 months. Findings showed that the mean survival time was longer in those without malnutrition risk [171.9 (167.1–176.9) days vs. 153.1 (140.0–166.2) days]. In the adjusted Cox regression model, the risk of death was associated with the risk of malnutrition, older age, and a high burden of comorbidities. In light of these data, the authors concluded the importance of paying attention to malnutrition to initiate early interventions in this subset of the elderly population.

Zhou et al. conducted research to investigate the metabolic basis of the cognitive improvement driven by active B-vitamin supplementation by carrying out an extensive metabolomic analysis of 302 metabolites identified in serum samples at baseline and at 24 months of a cohort of 137 subjects randomly assigned to active supplementation or placebo. Pathway analysis showed increased gluconeogenesis and War-burg effects underlying cognitive improvement in non-aspirin-using subjects supplemented with active B vitamins. Furthermore, metabolomics revealed that aspirin use may interact with B vitamin supplementation by altering gut microbial metabolism, particularly in terms of propionate production. Finally, omics data showed that differing ability to assimilate B vitamins at baseline, perhaps mediated by differences in gut microbial composition, may underlie variations in interindividual responses to active B vitamin supplementation.

Liao et al. investigated the predictive validity of the geriatric nutritional risk index (GNRI) in critically ill elderly patients with acute kidney injury. In this research, 1-year mortality was considered the primary outcome, while in-hospital, intensive care unit (ICU), 28- and 90-day mortality, and prolonged ICU and hospital length of stay were selected as secondary outcomes. Multivariable regression analysis identified the independent prognostic ability of GNRI on research outcomes. The restricted cubic spline showed a linear correlation between GNRI and death at 1 year. The prognostic implication of GNRI on 1-year mortality was still significant in patients with most subgroups. The authors therefore concluded that in critically ill elderly patients with acute kidney injury, an elevated GNRI at admission was strongly correlated with a lower risk of adverse outcomes.

Among the noteworthy review articles published in this Research Topic, we must mention the investigations by Abedi et al. and Rondanelli et al. Here, Abedi et al. conducted an interesting updated systematic review and meta-analysis to evaluate the efficacy of saffron supplementation on a cluster of oxidative stress markers in randomized controlled trials (RCTs). Saffron consumption was found to cause a significant decrease in malondialdehyde and total oxidant status and a significant increase in total antioxidant capacity levels. Subgroup analysis showed a significant reduction in malondialdehyde levels in studies with saffron dosage >30 mg/day, age <50 years, and study duration <12 weeks. Although the majority of Iranian studies could have been a limitation of the study, the results showed that saffron has beneficial effects on oxidative stress markers. Instead, Rondanelli et al. research group conducted a review with the goal of evaluating the most recent research on the ideal dietary approach to prevent or promote the treatment of diabetic retinopathy, age-related macular degeneration, and cataracts, as well as constructing a food pyramid that would make it easy for people at risk of developing these diseases to decide what to eat. The food pyramid presented proposed what should be consumed every day: 3 servings of low glycemic index (GI) cereals (for fiber and zinc content), 5 servings (each serving: ≥200 g/day) of fruits and vegetables (spinach, broccoli, cooked zucchini, green leafy vegetables, oranges, kiwis, grapefruits are preferred for the content of folic acid, vitamin C and lutein/zeaxanthin, at least ≥42 μg/day), extra virgin olive oil (almost 20 mg/day for the content of vitamin E and polyphenols), nuts or oilseeds (20–30 g/day, for the content of zinc, at least ≥15.8 mg/day); weekly: fish (4 servings, for omega-3 content and eicosapentaenoic acid (EPA) + docosahexaenoic acid (DHA) 0.35–1.4 g/day), white meat (3 servings, for vitamin B12 content), legumes (2 servings, for vegetable protein), eggs (2 servings, for lutein/zeaxanthin content), light cheeses (2 servings, for vitamin B6 content), and almost 3–4 times/week microgreens and spices (saffron and curcumin). At the top of the pyramid are two pennants: a green one, indicating the need for individualized supplementation (if daily requirements cannot be met by diet, supplementation of omega-3 and L-methylfolate), and a red one, indicating that certain foods are prohibited (salt and sugar). Finally, 30–40 minutes of aerobic and resistance exercise is required 3–4 times a week.

No less noteworthy, the present Collection included an analysis of the knowledge domain and emerging trends in nutrition research in sarcopenia (2, 7) and to provide implications for future research and strategies to prevent or manage sarcopenia in the context of an aging society. This research, conducted by Huang et al. was designed to provide health professionals and scholars with a comprehensive mapping of the knowledge base of nutrition and sarcopenia research over the past 30 years, as well as to help them quickly grasp research hot spots and choose future research projects.

Finally, Beasley et al. presented a randomized BRIDGE (BRInging the Diabetes prevention program to GEriatric Populations) study protocol aimed at comparing an in-person Diabetes Prevention Program (DPP) adapted for older adults (DPP-TOAT, i.e., Tailored for Older AdulTs) with a DPP-TOAT delivered via virtual group sessions (V-DPP-TOAT) in a randomized controlled trial design (*N* = 230). The primary efficacy outcome will be weight loss at 6 months and the primary implementation outcome will be participation in intervention sessions with a non-inferiority design. The results will inform best practices in the delivery of an evidence-based intervention.

In conclusion, when it comes to modifiable factors in the context of non-communicable diseases related to aging, nutrition remains critical. There remains ample room for research into the biological mechanisms underlying dietary strategies, individual foods, nutritional status, and dietary patterns that can shape the trajectories of chronic health outcomes, especially in the increasingly aging population.

## Author contributions

RZ: Writing – original draft. FC: Data curation, Methodology, Software, Writing – original draft. HC: Visualization, Writing – review & editing. GD: Visualization, Writing – review & editing. MC: Methodology, Supervision, Writing – review & editing. RS: Conceptualization, Supervision, Writing – review & editing.
